# Thrombus or Tumor: Thrombocytopenia-Led Diagnosis of Cardiac Sarcoma

**DOI:** 10.7759/cureus.99163

**Published:** 2025-12-13

**Authors:** Soomal Rafique, Amit Bhandari, Prakash Raj Oli, Priya Antil, Avinash Murthy

**Affiliations:** 1 Department of Internal Medicine, Southern Illinois University School of Medicine, Springfield, USA; 2 Department of Internal Medicine, HSHS St. John's Hospital, Springfield, USA; 3 Department of Internal Medicine, Karnali Provincial Hospital, Birendranagar, NPL; 4 Department of Cardiology, Prairie Heart Institute, Springfield, USA

**Keywords:** cardiac mass, cardiac sarcoma, cardiac tumor, pulmonary embolism, thrombocytopenia

## Abstract

Cardiac tumors, including benign myxomas and primary malignant tumors, are very rare compared with metastatic tumors. Thrombocytopenia, though rare, has been associated with cardiac tumors such as lymphoma and myxoma, potentially due to mechanical forces, platelet activation from turbulent blood flow, or autoimmune responses. We present a case of an elderly male who presented with shortness of breath and new-onset thrombocytopenia and was later found to have pleomorphic sarcoma. Following surgery, the patient’s platelet counts normalized, and respiratory symptoms improved. This case is noteworthy due to the rare combination of a cardiac mass, bilateral pulmonary embolism, and severe thrombocytopenia, which posed significant diagnostic and therapeutic challenges. It highlights a potential association between cardiac sarcoma and thrombocytopenia and underscores the diagnostic complexities involved in managing cardiac masses. The steady improvement in platelet count following sarcoma resection suggests a possible pathophysiological connection between the two conditions.

## Introduction

Cardiac tumors are exceptionally rare, with a prevalence ranging from 0.0017% to 0.33% [1]. Primary cardiac tumors are particularly uncommon compared with metastatic cardiac tumors, which occur 20-30 times more frequently [2]. Of all primary cardiac tumors, approximately 85% are benign, and 15% are malignant [2]. In adults, myxomas are the most common benign tumors, accounting for 40-70% of primary cardiac tumors, followed by papillary fibroelastomas, whereas in children, rhabdomyomas predominate, representing over 60% of pediatric cardiac tumors [2]. Most primary malignant tumors are sarcomas [1]. Patients with cardiac sarcoma often exhibit nonspecific symptoms such as dyspnea, chest pain, fever, malaise, and weight loss. Occasionally, these tumors may embolize, causing pulmonary or systemic embolism depending on their location [3]. Rarely, thrombocytopenia has also been reported in association with cardiac tumors [4-6]. We present a rare case of right-sided cardiac sarcoma causing severe thrombocytopenia and pulmonary embolism (PE).

## Case presentation

A 75-year-old man with a history of bilateral simple renal cysts was referred to the emergency department by his hematologist for evaluation of thrombocytopenia. He reported worsening shortness of breath and easy fatigue over six weeks. His initial platelet count was 88,000/µL, which dropped to 39,000/µL on repeat testing. His symptoms worsened, including severe fatigue with minimal activity, but he reported no chest or leg pain. Initial vital signs included a blood pressure of 134/78 mmHg, a heart rate of 84 beats per minute, a respiratory rate of 20 breaths per minute, and pulse oximetry showed oxygen saturation of 90%. ECG revealed a sinus rhythm at 73 beats per minute, and arterial blood gas analysis showed metabolic acidosis. Initial laboratory results are shown in Table 1.

**Table 1:** Pertinent laboratory workup

Lab parameter (unit)	Result	Normal values
Platelets (10³/µL)	32,000	150-400
International normalized ratio	1.6	0.8-1.1
D-dimer (ng/mL)	1440	0-500
Lactate dehydrogenase (U/L)	388	87-241
Fibrinogen (mg/dl)	117	200-393

**Figure 1:**
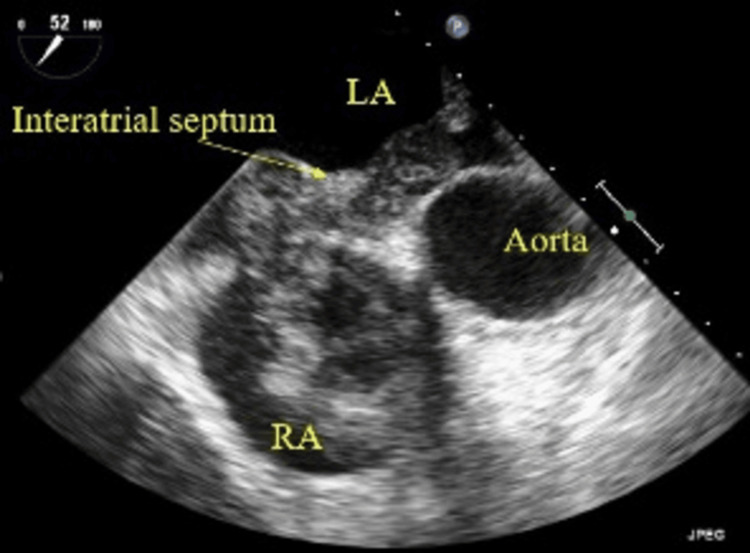
Transesophageal echocardiogram showing a mass in the RA LA, left atrium; RA, right atrium

Due to elevated D-dimer levels, the patient underwent chest CT angiography (CTA), which showed multiple bilateral submassive pulmonary artery filling defects and a large right atrium (RA) thrombus/mass extending into the right ventricle (RV). Compressed duplex venous ultrasonography of the inferior vena cava, bilateral iliac veins, and veins of the lower extremities did not reveal acute or chronic deep vein thrombosis. Transesophageal echocardiography (TEE) demonstrated a very large mass in the right heart extending from the RA into the RV, measuring 4.1 × 2.6 cm in the RV, with a moderately enlarged RV showing normal systolic function, a normal left ventricular ejection fraction of 55%, no patent foramen ovale, and an enlarged inferior vena cava without any mass or thrombus (Figure 1).

**Figure 2:**
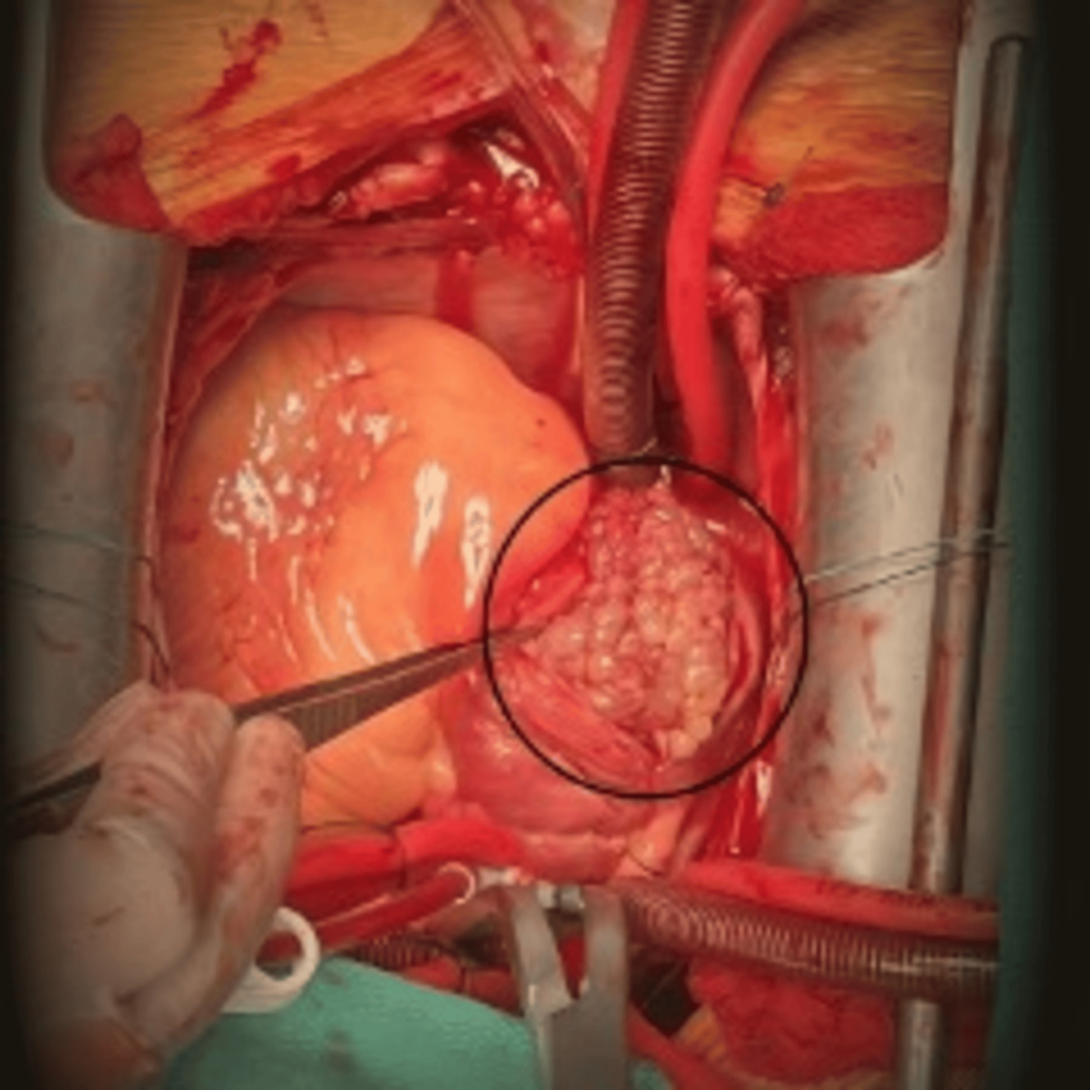
Intraoperative image showing a large, friable, verrucous-appearing mass

The combination of severe thrombocytopenia, a right heart mass, bilateral pulmonary artery filling defects on CTA, and elevated D-dimer raised concern for a right heart thrombus (thrombi-in-transit) with acute PE versus a right heart tumor (cardiac myxoma, papillary fibroelastoma, or lipoma) with tumor embolism to the lung or superimposed PE. The patient was admitted to the intensive care unit, and a heparin drip was initiated. Given the large size and mobility of the mass, along with worsening thrombocytopenia, emergency surgical intervention was performed. Surgery was conducted via standard median sternotomy with cardiopulmonary bypass. Intraoperatively, a large, friable, verrucous-appearing mass measuring 10.2 × 8.1 cm was noted, extending from the RA to the RV (Figure 2).

The mass was removed using a piecemeal technique. It had no distinct stalk but was densely adherent to the right atrial appendage, requiring the removal of a portion of the right atrial appendageal muscle along with the mass. Following resection, TEE showed no residual mass in the RA or RV; however, a 1.5 × 1.0 cm non-intraluminal mass was noted in the lower part of the interatrial septum. The frozen section suggested a right heart myxoma. However, histopathological examination revealed a predominantly papillary mass composed of sheets of spindle cells with hyperchromatic nuclei, marked pleomorphism, and conspicuous mitotic figures arranged in cords, with abundant myxoid stroma in a solid growth pattern, patchy areas of necrosis, and an adjacent admixed fibrin thrombus. Immunohistochemistry showed weak, patchy reactivity for FLI1, while the cells were negative for AE1/AE3, CD34, desmin, Sox10, and ALK. The right heart mass was therefore diagnosed as an undifferentiated pleomorphic sarcoma.

After surgical resection of the cardiac mass, the patient’s thrombocytopenia rapidly improved, normalizing by postoperative day 3. Postoperatively, he developed atrial fibrillation, which resolved spontaneously within 72 hours. His shortness of breath significantly improved after surgery. He was discharged home on anticoagulation with warfarin for bilateral PE secondary to malignancy and was referred to an oncologist for chemotherapy.

## Discussion

Cardiac tumors are extremely rare, with a prevalence of 0.0017% to 0.33% [1]. Most are benign myxomas, while primary malignant tumors are mainly sarcomas, such as angiosarcomas and undifferentiated sarcomas. Metastatic tumors to the heart, from sources such as lung, breast, or renal cancer, melanomas, lymphomas, and leukemias, are much more common, occurring 40 to 100 times more frequently than primary malignant cardiac tumors [1]. Undifferentiated pleomorphic sarcoma, an aggressive soft tissue sarcoma originating from mesenchymal stem cells, usually develops in the left atrium. Right heart sarcomas, which are more common in young adults, often present with nonspecific symptoms, making preoperative diagnosis challenging [7].

Patients with cardiac sarcoma frequently exhibit nonspecific symptoms such as dyspnea, chest pain, fever, malaise, and weight loss. Larger tumors can obstruct blood flow, disrupt valve function, cause arrhythmias due to local invasion, lead to pericardial effusion with tamponade, and trigger embolic events, including cerebral, coronary, and retinal emboli. Symptom severity varies based on tumor size, location, mobility, growth rate, friability, and invasiveness [3]. Compared with left heart and pulmonary artery sarcomas, right heart sarcomas tend to be bulky but less obstructive, demonstrate exophytic growth, are more infiltrative, and metastasize earlier [6]. Right-sided tumors can also embolize to cause PE, while left-sided tumors can lead to systemic embolism. Tissue fragmentation, detachment of tumor, and/or dissemination of overlaying thrombi or tumor surface foci are potential mechanisms responsible for embolism in cardiac tumors [8].

Thrombocytopenia is a rare finding reported in association with cardiac tumors. It has been described in various solid tumors, including cardiac lymphoma [4], myxoma [5], and cardiac synovial sarcoma [6]. In these cases, thrombocytopenia occurred either as an isolated hematologic abnormality or in conjunction with anemia or erythrocytosis, and platelet counts improved after tumor resection. Although the precise mechanism behind thrombocytopenia in cardiac tumors remains unclear, it is thought to result from mechanical forces exerted by the tumor, platelet activation and consumption due to turbulent blood flow, or the tumor’s potential to trigger autoimmune platelet destruction [9]. Other proposed mechanisms include drug-induced thrombocytopenia, disseminated intravascular coagulation (DIC), thrombotic thrombocytopenic purpura, paraneoplastic syndrome, and idiopathic thrombocytopenic purpura [10].

To identify the cause of thrombocytopenia, extensive testing ruled out multiple factors: nutritional and infectious causes (e.g., vitamin B12, folate, HIV, hepatitis, and parvovirus), splenic platelet sequestration (normal-sized spleen on CT), and medication-related thrombocytopenia (no recent heparin use). Autoimmune tests (anti-phospholipid, anti-systemic lupus erythematosus, and antiplatelet antibodies) were negative, making antibody-induced thrombocytopenia unlikely. Ultrasound excluded deep vein thrombosis, and there were no signs of immune or thrombotic thrombocytopenia (no end-organ damage or microangiopathic hemolytic anemia). Normal white blood cell and hemoglobin levels suggested intact platelet production. However, the patient had mildly elevated LDH and bilirubin, decreased haptoglobin, reduced fibrinogen, and elevated D-dimer with mild coagulopathy, raising the possibility of DIC or cancer-associated thrombotic microangiopathy. DIC, affecting 10-15% of cancer patients, can present in procoagulant, hyperfibrinolytic, or subclinical forms. Most solid tumors fall into the subclinical category, except for mucin-producing tumors such as pancreatic and lung adenocarcinomas [11]. In this case, the patient appeared to have a subclinical form of DIC, and notably, platelet counts normalized after resection of the cardiac sarcoma, suggesting a potential association between thrombocytopenia and cardiac sarcoma.

This case is notable for the rare combination of a cardiac mass, bilateral PE, and severe thrombocytopenia, which posed diagnostic and therapeutic challenges. The initial CTA of the chest could not distinguish between a cardiac mass and a thrombus. Transthoracic echocardiography suggested a cardiac mass, leading to a preliminary diagnosis of cardiac myxoma. While TEE is highly precise and cost-effective for assessing intracardiac masses, it struggles to differentiate between benign and malignant tumors. Cardiac MRI (CMR), CT, and PET/CT can assist with differential diagnosis, including evaluating extracardiac extent and distant metastases if the mass is tumorous. Coronary angiography is often performed to assess tumor invasion of the coronary arteries. CMR provides exceptional visualization of cardiac anatomy, tissue characteristics, and perfusion, whereas CT is useful for assessing the lungs, pleura, and mediastinal involvement [9].

The prevalence of cardiac tumors, clinical presentation, and demographic patterns of patients do not appear to vary substantially by race; however, mortality patterns show notable racial disparities, with African Americans demonstrating consistently higher age-standardized mortality rates from cardiac neoplasms compared with Whites during the 2000-2019 period [12]. Cardiac sarcomas carry a poor prognosis, with a median survival of approximately one year. Complete surgical resection remains the key treatment and the only intervention proven to improve survival [7]. The benefits of adjuvant systemic therapies after surgery for cardiac sarcomas are unclear due to the rarity of the condition and the lack of randomized trials. Nevertheless, adjuvant chemotherapy and radiotherapy are recommended for advanced, recurrent, or persistently positive-margin tumors, as surgery alone may be insufficient [3].

## Conclusions

This case highlights a rare and noteworthy association between cardiac sarcoma and severe thrombocytopenia, emphasizing the diagnostic complexities that cardiac masses often present. The rapid and sustained improvement in platelet count following surgical resection of the sarcoma suggests a potential pathophysiological connection between the tumor and the hematologic abnormality, possibly involving mechanisms such as tumor-related platelet consumption, immune-mediated destruction, or intravascular activation of coagulation. Although advanced multimodal imaging, including echocardiography, CT, and MRI, can provide valuable anatomical and functional information, this case illustrates that such modalities may not reliably distinguish malignant from benign cardiac lesions. Definitive diagnosis required histopathological evaluation, which remains the gold standard for characterizing cardiac tumors. Given the rarity of thrombocytopenia in the context of cardiac malignancy, additional case reports and systematic studies are needed to better elucidate the underlying mechanisms and to guide future diagnostic and therapeutic strategies.
